# Heat shock protein 60 regulates yolk sac erythropoiesis in mice

**DOI:** 10.1038/s41419-019-2014-2

**Published:** 2019-10-10

**Authors:** Yaoyun Duan, Hong Wang, Kalia Mitchell-silbaugh, Shangbin Cai, Feifei Fan, Yali Li, Huayuan Tang, Gang Wang, Xi Fang, Jie Liu, Nan Jia, Ran Jing, Kunfu Ouyang

**Affiliations:** 1 School of Chemical Biology and Biotechnology, State Key Laboratory of Chemical Oncogenomics, Peking University Shenzhen Graduate School, 518055 Shenzhen, China; 2 Department of Medicine, School of Medicine, University of California San Diego, La Jolla, CA 92093 USA; 3 Department of Pathophysiology, School of Medicine, Shenzhen University, 518055 Shenzhen, China; 4 Shenzhen People’s Hospital, 518055 Shenzhen, China; 5 Department of Cardiovascular Medicine, Xiangya Hospital, Central South University, 410011 Changsha, China

**Keywords:** Apoptosis, Embryology

## Abstract

The yolk sac is the first site of blood-cell production during embryonic development in both murine and human. Heat shock proteins (HSPs), including HSP70 and HSP27, have been shown to play regulatory roles during erythropoiesis. However, it remains unknown whether HSP60, a molecular chaperone that resides mainly in mitochondria, could also regulate early erythropoiesis. In this study, we used Tie2-Cre to deactivate the *Hspd1* gene in both hematopoietic and vascular endothelial cells, and found that *Tie2-Cre*^+^*Hspd1*^f/f^ (HSP60^CKO^) mice were embryonic lethal between the embryonic day 10.5 (E10.5) and E11.5, exhibiting growth retardation, anemia, and vascular defects. Of these, anemia was observed first, independently of vascular and growth phenotypes. Reduced numbers of erythrocytes, as well as an increase in cell apoptosis, were found in the HSP60^CKO^ yolk sac as early as E9.0, indicating that deletion of HSP60 led to abnormality in yolk sac erythropoiesis. Deletion of HSP60 was also able to reduce mitochondrial membrane potential and the expression of the voltage-dependent anion channel (VDAC) in yolk sac erythrocytes. Furthermore, cyclosporine A (CsA), which is a well-recognized modulator in regulating the opening of the mitochondrial permeability transition pore (mPTP) by interacting with Cyclophilin D (CypD), could significantly decrease cell apoptosis and partially restore VDAC expression in mutant yolk sac erythrocytes. Taken together, we demonstrated an essential role of HSP60 in regulating yolk sac cell survival partially via a mPTP-dependent mechanism.

## **I**ntroduction

In both mice and humans, hematopoietic development occurs in several waves and at different anatomic sites, and a range of different blood cell types are generated during embryogenesis^1,2^. It has been shown that the first wave of hematopoietic progenitors, or primitive hematopoiesis, in mice emerges in “blood islands” in the yolk sac on embryonic day 7.5 (E7.5). This results in the production of the first circulating blood cells of erythroid, megakaryocyte, and macrophage lineages^3,4^. Primitive erythroid cells enter the circulation at E8.5 to E9.0^5^. At E8.25−8.5, a definitive hematopoietic program also starts in the yolk sac with erythromyeloid progenitors, which further colonize in the fetal liver prior to hematopoietic stem cells^6,7^. While erythromyeloid progenitors are transient, they serve as a critical source of fetal hematopoiesis before hematopoietic stem cells expand and differentiate^8^. Moreover, erythromyeloid progenitors could also differentiate into endothelial cells, and thus promote the formation of the yolk sac endothelium and modulate embryonic vascular remodeling^9,10^. At around E10.0, a third wave of hematopoietic potential, consisting of adult repopulating hematopoietic stem cells, emerges from the aorta-gonad-mesonephros region^1^. However, how hematopoietic potentials change so rapidly in such a narrow developmental window and the molecular mechanisms underlying how different hematopoiesis stages are regulated have not yet been well established.

Heat shock proteins (HSPs) are divided into different families according to molecular weight and amino acid sequence homology, including HSP110/100, HSP90, HSP70, HSP60, HSP40, and small HSPs^11,12^. As molecular chaperones, it has been shown that HSPs could serve multiple functions in regulating erythropoiesis^13^. For example, in Diamond-Blackfan anemia, chaperone activity has been suggested to play a role in optimizing protein synthesis in immature erythroblasts^14^. It has also been shown that HSP70 regulates erythropoiesis by preventing Caspase-3-mediated cleavage of GATA1^15^. HSP27 is phosphorylated by p38 and then enters the nucleus and binds to GATA1, inducing the ubiquitination and proteasomal degradation of the latter^16^. In mitochondria, HSP60 forms a large multi-subunit complex with its co-chaperonin HSP10 to facilitate proper protein folding^11^, and has been shown to play an essential role in the survival of yeast, Drosophila, and mice^17–19^. However, it remains unknown whether HSP60 could play a role in regulating erythropoiesis, since deletion of HSP60 in mice results in an early embryonic lethality shortly after implantation (E6.5 to E7.5), when “blood islands” have not yet occurred in the yolk sac^17^. In this study, we used Tie2-Cre in mice to delete the *Hspd1* gene in both hematopoietic and vascular endothelial cells, and revealed an essential role of HSP60 in regulating yolk sac erythropoiesis.

## Results

### Deletion of HSP60 by Tie2-Cre resulted in anemia and embryonic lethality

We first examined the expression of HSP60 in wildtype yolk sac erythrocytes at E9.0 using immunofluorescence staining. The erythrocytes were labeled with GATA1, a transcription factor which has been shown to play an essential role in erythromegakaryocytic differentiation and has been widely used as a sensitive and specific marker for erythroid and megakaryocytic lineages^20,21^. The voltage-dependent anion channel (VDAC) and Cytochrome C (Cyt C) are two mitochondrial proteins commonly used to label the distribution of mitochondria inside cells^22,23^. Using an antibody recognizing all three isoforms of mammalian VDACs and another antibody recognizing Cyt C, we found that both exhibited strong and clear perinuclear distribution (Supplemental Fig. 1a, b). Consistently, HSP60, which is generally recognized as one of the mitochondrial molecular chaperones^24^, also showed a similar distribution pattern as VDAC and Cyt C in E9.0 wildtype yolk sac erythrocytes (Supplemental Fig. 1c).

**Fig. 1:**
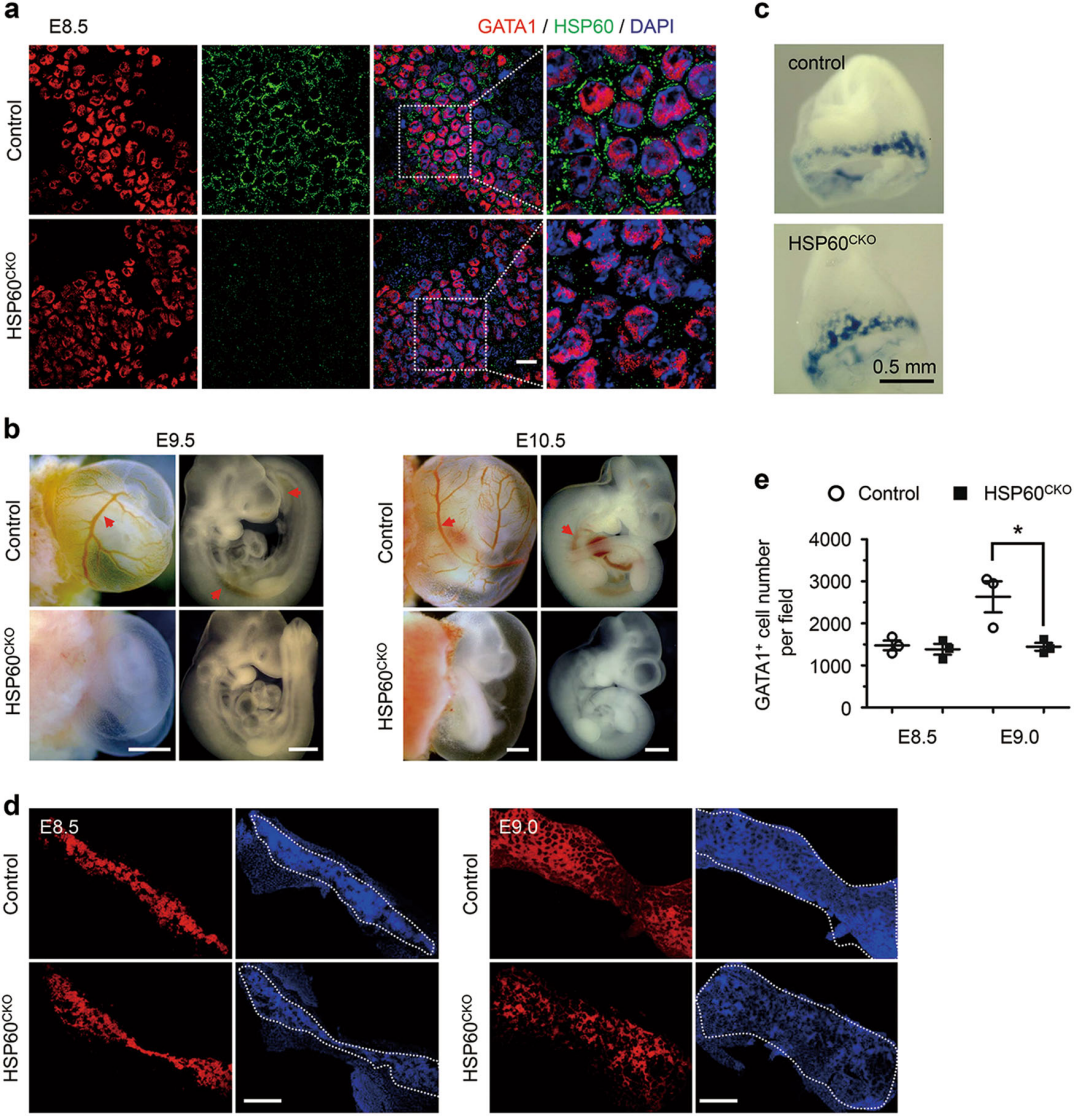
Deletion of HSP60 by Tie2-Cre in mice resulted in reduced numbers of yolk sac erythrocytes and anemia. **a** Whole-mount immunofluorescence staining was used to examine the expression of HSP60 in control and HSP60^CKO^ yolk sac erythrocytes at E8.5. Data are representative of at least three independent experiments. Scale bar, 20 μm. **b** Morphology of control and HSP60^CKO^ yolk sacs and embryos isolated at E9.5 and E10.5, indicating that red blood cells (red arrows) can be easily observed in control yolk sac vitelline vessels and embryo dorsal aorta at both stages, whereas HSP60^CKO^ yolk sacs and embryos appear extremely pale. Scale bar, 0.5 mm. **c** Embryos with yolk sacs at E8.5 were stained with benzidine, which stains all hemoglobin-containing cells blue. **d** Whole-mount GATA1 immunofluorescence staining was used to calculate the numbers of erythrocytes in the whole yolk sacs at E8.5 and E9.0, respectively. Each picture was stitched with 3–4 individual images captured on the same yolk sac. Scale bar, 0.5 mm. **e** Numbers of GATA1 positive cells were calculated in control and HSP60^CKO^ yolk sacs at E8.5 and E9.0, respectively. *n* = 4 mice per group. All data represent mean ± SEM. Significance was determined using a 2-tailed, unpaired Student’s *t* test. *p < 0.05 versus control

To investigate the physiological function of HSP60 in erythropoiesis, we crossed *Hspd1*^*f*/f^ mice with *Tie2-Cre*^+^ mice that express the Cre recombinase under the control of the mouse endothelial-specific receptor tyrosine kinase (Tie2) promoter^25^. Tie2-Cre has been shown to target both endothelial and blood cells in the blood islands in early developing yolk sacs^26^. *Tie2-Cre*^+^*Hspd1*^f/+^ mice were fertile with no obvious phenotype, and were further used to cross with *Hspd1*^f/f^ mice to generate *Tie2-Cre*^+^*Hspd1*^f/f^ (HSP60^CKO^) mice (Supplemental Fig. 2a). Polymerase chain reaction was used to confirm the genotypes of control and HSP60^CKO^ mice (Supplemental Fig. 2b). Furthermore, immunofluorescence staining showed that HSP60 also exhibited a clear perinuclear localization in GATA1 positive erythrocytes in control yolk sacs at E8.5, which was absent in HSP60^CKO^ yolk sacs at the same stage (Fig. 1a), suggesting that deletion of HSP60 by Tie2-Cre was very efficient in early developing erythrocytes.

Importantly, no live HSP60^CKO^ mice were observed at the postnatal day 10 (P10; Table 1), and thus timed-pregnant matings were carried out to determine whether HSP60^CKO^ mice were embryonic lethal. All HSP60^CKO^ embryos observed between E11.5 and E13.5 were dead and absorbed. At E10.5, HSP60^CKO^ embryos could be observed at the expected Mendelian ratio (Table 1), but all mutant embryos were dramatically growth retarded and developed severe anemia. The latter was demonstrated by a pale appearance and an abnormally low number of blood cells in either embryos or yolk sacs (Fig. 1b). No obvious growth retardation could be detected in HSP60^CKO^ embryos compared with control embryos at E9.5, but red blood cells were difficult to observe in mutant yolk sacs and embryos (Fig. 1b).

**Table 1:** Genotypic analysis of embryos from *Tie2-Cre*^+^*Hsp60*^f/+^ × *Hsp60*^f/f^ intercrosses

Genotype age	Tie2-Cre^−^ Hsp60^f/+^	Tie2-Cre^−^ Hsp60^f/f^	Tie2-Cre^+^ Hsp60^f/+^	Tie2-Cre^+^ Hsp60^f/f^	Total
E8.5	49 (25.9)	48 (25.4)	51 (27.0)	41 (21.7)	189
E9.5	26 (18.4)	35 (24.8)	43 (30.5)	37 (26.2)^a^	141
E10.5	13 (21.0)	19 (30.6)	15 (24.2)	15 (24.2)^b^	62
E11.5	14 (35.9)	7 (17.9)	10 (25.6)	8 (20.5)^c^	39
E12.5	3 (30.0)	3 (30.0)	3 (30.0)	1 (10.0)^c^	10
E13.5	7 (24.1)	12 (41.4)	6 (20.7)	4 (13.8)^c^	29
P10	36 (39.6)	26 (28.6)	29 (31.9)	0 (0.0)	91
Expected (%)	25.0	25.0	25.0	25.0	100

^a^All embryos are pale

^b^All embryos are pale and growth retarded

^c^All embryos are dead and almost absorbed

We then examined whether deletion of HSP60 could influence the erythropoiesis in yolk sacs. First, benzidine staining showed that blood distribution in yolk sacs was comparable between control and HSP60^CKO^ mice at E8.5 (Fig. 1c). Consistently, the numbers of yolk sac erythrocytes calculated from whole-mount GATA1 immunostaining were also comparable between control and HSP60^CKO^ mice at the same stage (Fig. 1d, e). However, the numbers of yolk sac erythrocytes in HSP60^CKO^ mice at E9.0 were significantly reduced when compared with control mice (Fig. 1d, e).

### HSP60^CKO^ embryos developed late-onset vascular abnormalities

**Fig. 2:**
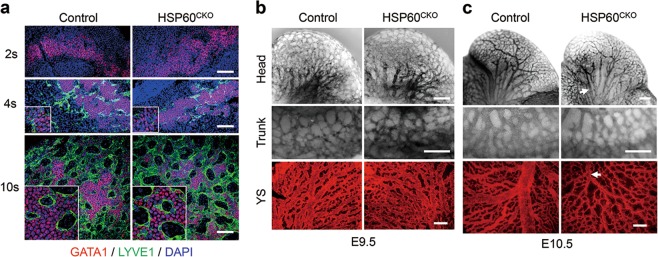
Endothelial cell specification and vascular development in control and HSP60^CKO^ mice. **a** Immunofluorescence staining of GATA1 and LYVE1 in yolk sacs isolated at the 2-somite (2 s), 4-somite (4 s), and 10-somite (10 s) ages, respectively. Data are representative of at least three independent experiments. Scale bar, 100 μm. **b** and **c** Vasculature in control and HSP60^CKO^ embryonic heads (top), trunks (middle), and yolk sacs (bottom) identified by PECAM1 staining at E9.5 (**b**) and E10.5 (**c**). Blood vessels with reduced diameters (white arrows) were easily observed in HSP60^CKO^ samples at E10.5. Data are representative of at least three independent experiments. Scale bar, 200 μm

Considering that both endothelial cells and erythrocytes in early developing yolk sacs may come from a common erythromyeloid progenitor^9,10^, and that Tie2-Cre contributed to both endothelial and blood cell lineages^26^, we then investigated the effects of HSP60 deletion by Tie2-Cre on endothelial and vascular development. Lymphatic vessel endothelial hyaluronan receptor 1 (LYVE1), which is generally recognized as a lymphatic endothelial cell marker, was also used as an endothelium marker in yolk sac blood vessels during embryonic development^27^. In control yolk sacs, GATA1 positive, but not LYVE1 positive, cells could be detected at the 2-somite stage. Soon after, a subset of GATA1 positive cells started to express LYVE1 at the 4-somite stage. Later on, these GATA1 and LYVE1 double positive endothelial cells were further differentiated, losing GATA1 expression, and formed many honeycomb-like vessel structures in yolk sacs at the 10-somite stage. However, no apparent changes in the timing of expression or the patterning of LYVE1 could be found in HSP60^CKO^ yolk sacs when compared with control yolk sacs at all stages. In addition, the formation of honeycomb-like structures was also typical in HSP60^CKO^ yolk sacs (Fig. 2a). The results all suggest that the deletion of HSP60 has no major influence on endothelial cell differentiation or early development of the yolk sac vascular system.

We also performed whole-mount PECAM1 staining to investigate the effects of HSP60 deletion on embryonic and yolk sac vascular development at later stages. At E9.5, the major profiles of the vasculature system including vascular patterning, vessel density, and vessel diameter were not significantly altered in the HSP60^CKO^ head, trunk, and yolk sac (Fig. 2b). However, some mutant vessels were easily observed with reduced diameters in the mutant embryonic head, trunk, and yolk sac when compared with control vessels in the same location at E10.5 (Fig. 2c).

### *HSP60* deficiency increased cell apoptosis of yolk sac erythrocytes

**Fig. 3:**
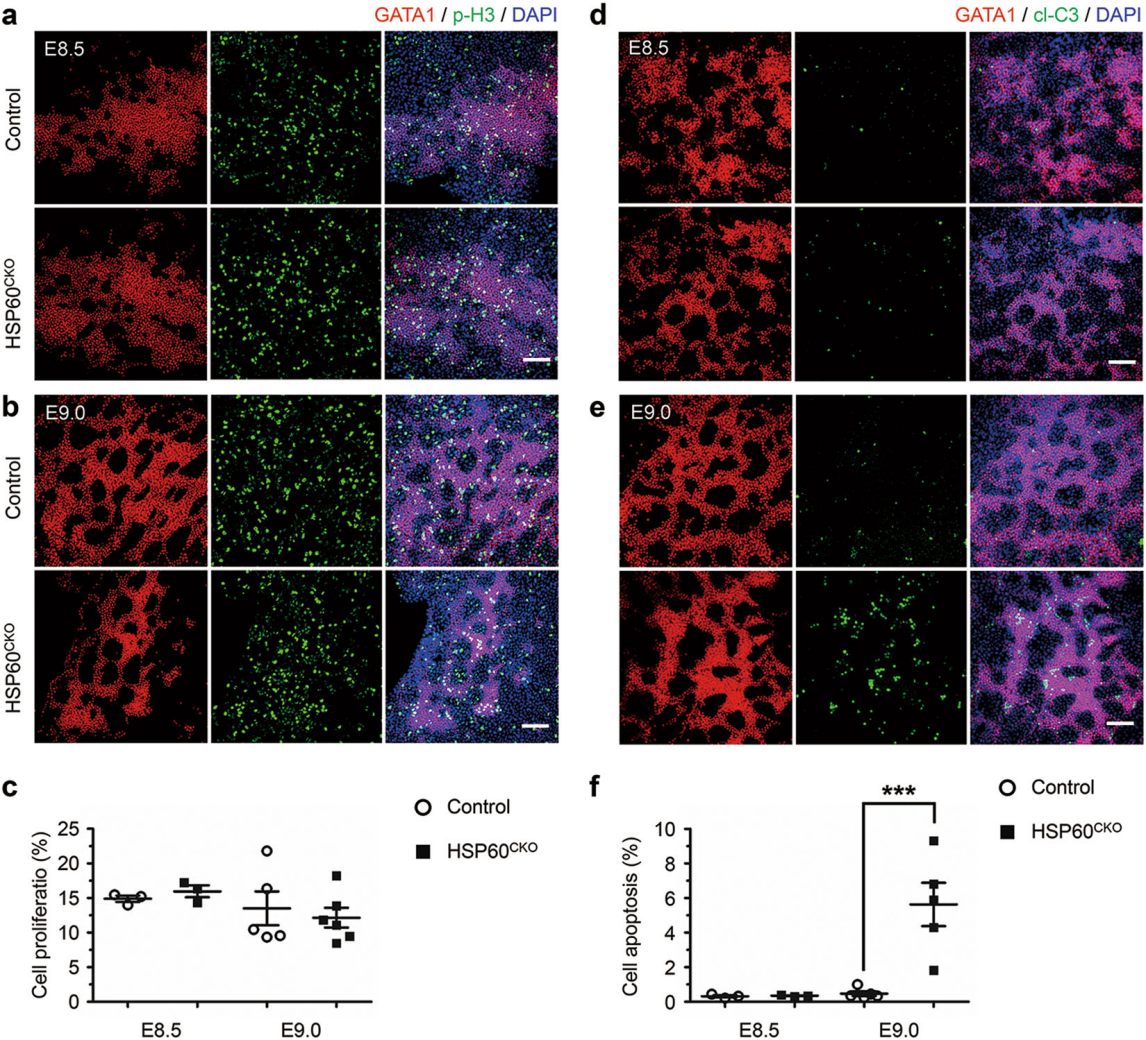
Deletion of Hsp60 increased cell apoptosis in erythrocytes. **a**, **b** Immunofluorescence staining of GATA1 and phosphorylated Histone 3 (p-H3) in control and HSP60^CKO^ yolk sacs at E8.5 (**a**) and E9.0 (**b**), respectively. Scale bar, 100 μm. **c** Statistical analysis showing that the numbers of p-H3 and GATA1 double positive cells are not significantly altered in HSP60^CKO^ yolks (*n* = 3, E8.5; *n* = 6, E9.0) at both ages compared with control yolk sacs (*n* = 3, E8.5; *n* = 5, E9.0). All data represent mean ± SEM. Significance was determined using a 2-tailed, unpaired Student’s *t* test. **d** and **e** Immunofluorescence staining of GATA1 and cleaved Caspase3 (cl-C3) in control and HSP60^CKO^ yolk sacs at E8.5 (**d**) and E9.0 (**e**), respectively. Scale bar, 100 μm. **f** Statistical analysis showing that the numbers of cl-C3 and GATA1 double positive cells are significantly reduced in HSP60^CKO^ yolk sacs (*n* = 3, E8.5; *n* = 5, E9.0) at E9.0 compared with control yolk sacs (*n* = 3, E8.5; *n* = 5, E9.0). All data represent mean ± SEM. Significance was determined using a 2-tailed, unpaired Student’s *t* test. ***p < 0.001 versus control

We next investigated whether the decreased numbers of erythrocytes and the anemia observed in HSP60^CKO^ embryos were due to reduced cell proliferation or increased cell apoptosis. We isolated yolk sacs from control and HSP60^CKO^ embryos at E8.5 and E9.0, and performed whole-mount immunofluorescence staining to check cell proliferation and cell apoptosis, respectively. At both E8.5 and E9.0, cell proliferation in GATA1 positive erythrocytes was not significantly altered in HSP60^CKO^ yolk sacs, evidenced by comparable ratios of phosphorylated Histone 3 positive erythrocytes in control and mutant yolk sacs at both stages (Fig. 3a–c). On the other hand, cell apoptosis indicated by cleaved Caspase 3 positive staining was not significantly altered at E8.5, but was dramatically increased at E9.0 in mutant yolk sac erythrocytes when compared with control cells (Fig. 3d–f). Such an increase in cell apoptosis of yolk sac erythrocytes might lead to reduced numbers of erythrocytes and eventually resulted in anemia in mutant embryos at later stages.

### Deletion of *HSP60* perturbed mitochondrial membrane potential and VDAC expression

**Fig. 4:**
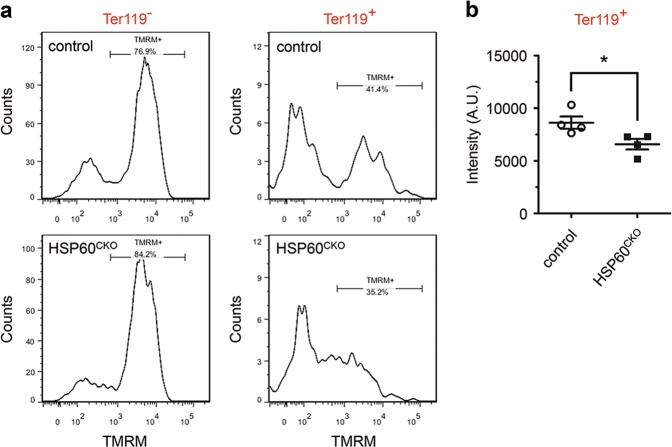
Deletion of HSP60 reduced mitochondrial membrane potential in yolk sac erythrocytes. Single cells were prepared from control and HSP60^CKO^ yolk sacs at E8.5, and flow cytometry was performed to measure mitochondrial membrane potential using tetramethylrhodamine (TMRM) in Ter119 positive (Ter119^+^) and Ter119 negative (Ter119^−^) cells. **a** Distribution of TMRM fluorescence of Ter119^−^ and Ter119^+^ cells in control and HSP60^CKO^ yolk sac cells. **b** Statistical analysis showing reduced TMRM fluorescence in HSP60^CKO^ Ter119^+^ cells. *n* = 4 mice per group. All data represent mean ± SEM. Significance was determined using a 2-tailed, unpaired Student’s *t* test. *p < 0.05 versus control

We then investigated how deletion of HSP60 could increase cell apoptosis of erythrocytes and cause anemia during embryonic development. It has been shown that HSP60 in tumor cells could directly bind with Cyclophilin D (CypD), an important modulator conferring sensitivity of the opening of the mitochondrial permeability transition pore (mPTP) to Cyclosporin A (CsA)^28,29^. Downregulation of HSP60 in these cells reduced mitochondrial membrane potential and resulted in cell apoptosis^30^, suggesting that HSP60 might play a similar function as CsA, inhibiting the opening of mPTP via binding to CypD. Therefore, we isolated single cells from E8.5 control and HSP60^CKO^ yolk sacs, and performed flow cytometry to examine whether HSP60 deficiency could also affect the mitochondrial membrane potential of yolk sac erythrocytes. We found that the mitochondrial membrane potentials of mutant Ter119 positive cells were slightly but significantly lower than those of control cells (Fig. 4a, b).

**Fig. 5:**
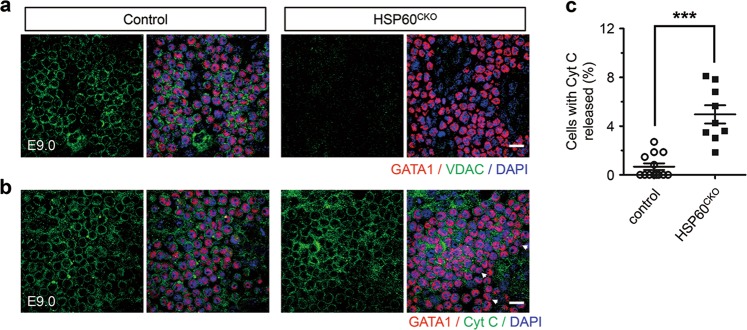
HSP60 deletion affected VDAC expression and Cyt C release in yolk sac erythrocytes. **a** Immunofluorescence staining of GATA1 and VDAC in control and HSP60^CKO^ yolk sacs at E9.0. HSP60 deletion abolished VDAC expression in yolk sac erythrocytes. Data are representative of at least three independent experiments. Scale bar, 20 μm. **b** Immunofluorescence staining of GATA1 and Cyt C in control and mutant yolk sacs at E9.0. The GATA1 positive cells with released Cyt C were characterized with no perinuclear immunofluorescence (white arrows). Data are representative of at least three independent experiments. Scale bar, 20 μm. **c** Statistical analysis showing increased numbers of the cells with released Cyt C in mutant erythrocytes. *n* = 13 from 5 control yolk sacs and 9 from 3 HSP60^CKO^ yolk sacs. All data represent mean ± SEM. Statistical significance was determined by Mann-Whitney *U* test. *p < 0.001 versus control

We also investigated whether HSP60 deletion affected the expression of particular mitochondrial proteins using immunofluorescence staining. To our surprise, deletion of HSP60 could significantly reduce VDAC expression in yolk sac erythrocytes at E9.0 when compared with control yolk sac erythrocytes at the same stage (Fig. 5a). We also sought to determine which VDAC isoforms were reduced in mutant cells. In mammals, VDAC has 3 distinct isoforms: VDAC1, VDAC2, and VDAC3^31^. Since only VDAC1^−/−^ mice with partial penetrance and VDAC2^−/−^ mice with 100% penetrance, but not VDAC3^−/−^ mice, developed embryonic lethality^32–34^, we focused more on the expression of VDAC1 and VDAC2. We found that VDAC2 was highly expressed in control yolk sac erythrocytes at E9.0, while VDAC1 was highly expressed in GATA1 negative cells. In addition, the expression of VDAC2 in mutant yolk sac erythrocytes was dramatically downregulated when compared with control cells at the same stage (Supplemental Fig. 3a, b). Furthermore, we also characterized the distribution of Cyt C in both control and HSP60^CKO^ erythrocytes. We found that most GATA1 positive cells exhibited clear circular and perinuclear Cyt C staining in both control and mutant yolk sacs at E9.0 (Fig. 5b). However, a subset of GATA1 positive cells appeared to have released Cyt C, indicated by no perinuclear immunofluorescence in these cells, which accounted for about 0.80 and 4.74% of total GATA1 positive cells in control and HSP60^CKO^ yolk sacs (Fig. 5c), respectively, which were similar to the rates of apoptotic cells observed in control and mutant yolk sacs, respectively as shown in Fig. 3, suggesting that deletion of HSP60 in yolk sac erythrocytes caused cell apoptosis via the mitochondrial pathway. On the other hand, we did not observe obvious changes in the expression of CypD between control and HSP60^CKO^ yolk sac erythrocytes (Supplemental Fig. 4a). We also examined expression of NIMP and COX4 in control and HSP60^CKO^ yolk sacs at the same stage. However, both these two proteins were highly expressed in GATA1 negative cells instead of in the erythrocytes (Supplemental Fig. 4b, c).

### Cyclosporin A partially rescued cell apoptosis of yolk sac erythrocytes induced by HSP60 deletion

**Fig. 6:**
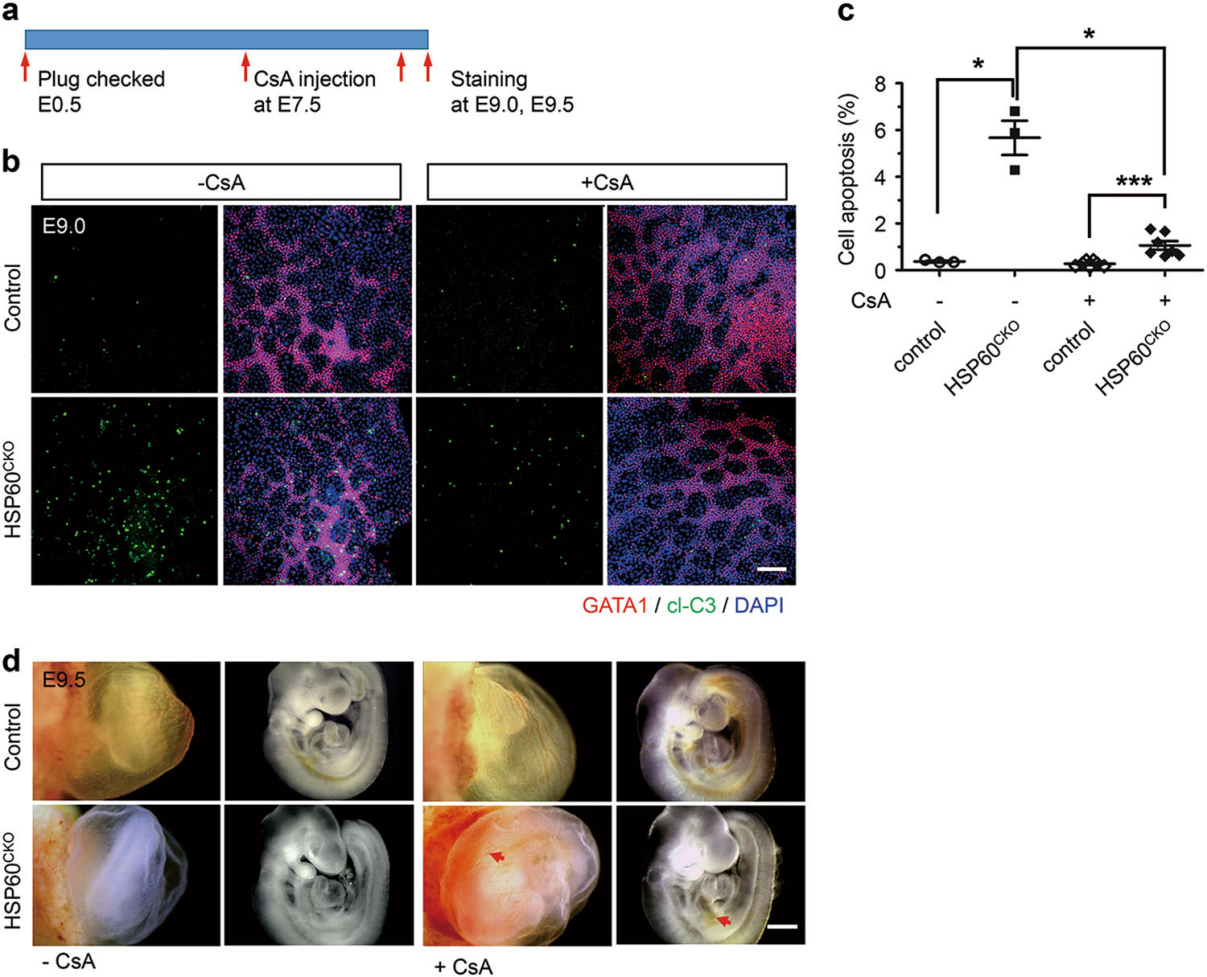
Cyclosporine A reduced cell apoptosis induced by HSP60 deletion in mutant erythrocytes. **a** Schematic diagram showing the procedure of the rescue experiment. Pregnant mice were intraperitoneally injected with cyclosporine A (CsA) at E7.5. The embryos were dissected at E9.0 or E9.5, and immunofluorescence staining was then performed. **b** Immunofluorescence staining of GATA1 and cleaved-Caspase 3 (cl-C3) in control and mutant yolk sacs treated with (+CsA) and without (−CsA) CsA at E9.0. Scale bar, 100 μm. **c** Statistical analysis showing that CsA treatment could significantly reduce cell apoptosis of erythrocytes in mutant yolk sacs (*n* = 3, −CsA; *n* = 7, +CsA) compared with control yolk sacs (*n* = 3, −CsA; *n* = 7, +CsA). Data represent mean ± SEM. Significance was determined using the 2-way ANOVA analysis with a Bonferroni post-hoc test. *p < 0.05, ***p < 0.001. **d** Morphology of control and mutant yolk sacs and embryos treated with CsA (+CsA) and without CsA (−CsA) at E9.5. Red arrows indicate vessels with red blood cells in mutant yolk sacs and embryos treated with CsA. Scale bar, 0.5 mm

We next investigated whether inhibition of caspases could block cell death of yolk sac erythrocytes. Pregnant mice were intraperitoneally injected with a single dose of Ac-DEVD-CHO, a potent inhibitor of Caspase 3/7/9, as well as other caspases^35^, or vehicle at 7.5 days after detection of a vaginal plug (Supplemental Fig. 5a), and cell apoptosis of yolk sac erythrocytes was then assessed at E9.0. We found that Ac-DEVD-CHO treatment could almost eliminate cell death of yolk sac erythrocytes, indicated by an ~90% reduction in cell apoptosis in HSP60^CKO^ yolk sac erythrocytes at E9.0 (Supplemental Fig. 5b, c). We further investigated the effect of CsA on cell survival of yolk sac erythrocytes after HSP60 deletion, and found that injection of CsA using the same protocol was also able to dramatically reduce cell apoptosis in HSP60^CKO^ yolk sac erythrocytes (Fig. 6a–c), even though the inhibition of cell apoptosis by CsA treatment (about 80%) was slightly lower than that by the direct caspase inhibitor. Consequently, more blood cells, evidenced by red patches, could be observed in both the trunk and the yolk sac in a subset of mutant embryos (3 of 10) treated with CsA when compared with those untreated with CsA (Fig. 6d). In addition, we found that CsA treatment could, at least partially, restore VDAC2 expression in mutant yolk sac erythrocytes at E9.0 (Supplemental Fig. 6). Taken together, these results demonstrated that treatment with the CypD inhibitor CsA could rescue both celluar and molecular phenotypes in mutant yolk sac erythrocytes.

## Discussion

**Fig. 7:**
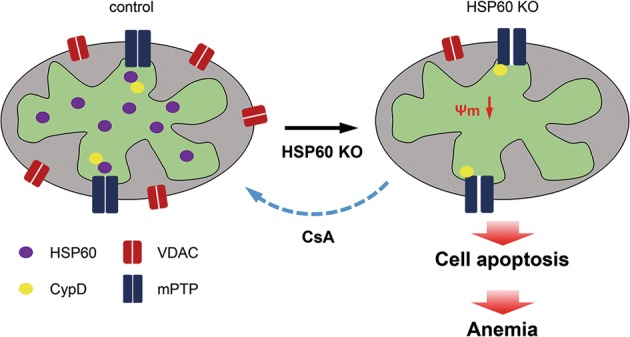
Schematic diagram showing the effects of HSP60 deletion in mouse yolk sac erythrocytes. In mouse yolk sac erythrocytes, deletion of HSP60 reduced mitochondrial membrane potential. In control cells, HSP60 might bind with CypD, preventing the latter from interacting with the mPTP. When HSP60 was deleted, CypD was liberated and bound to the mPTP, which thus increased the opening of the mPTP, decreased mitochondrial membrane potential, and induced more cell apoptosis. In the end, the numbers of yolk sac erythrocytes were reduced, and the mutant embryos developed anemia and died

In this study, we demonstrated that HSP60 plays an essential role in regulating cell survival of erythrocytes in the yolk sac (Fig. 7). In the developing mouse embryo, erythropoiesis first occurs from the so-called “blood islands” that are formed in the yolk sac at E7.5, producing red blood cells, macrophage, and megakaryocytes^36^. Previous studies have shown that the vascular endothelial growth factor (VEGF)/VEGF receptor 2 (VEGFR2 or FLK1) signaling axis is essential for the formation of the yolk sac blood islands. Failure of blood island formation was found in FLK^−/−^ and VEGF^+/−^ mice^37,38^. However, our results revealed that GATA1 positive erythrocytes in control and HSP60^CKO^ yolk sacs at E8.5 were still comparable, suggesting that deletion of HSP60 has no major effect on the formation of blood islands in the yolk sac. Moreover, deletion of HSP60 did not affect cell proliferation of yolk sac erythrocytes. By contrast, HSP60 deficiency significantly increased cell apoptosis of yolk sac erythrocytes, leading to anemia and a reduction in the numbers of yolk sac erythrocytes. Our results also suggest that deletion of HSP60 does not affect early endothelial cell differentiation and vasculature formation. Considering that the maintenance of blood viscosity and shear stress is required for normal vascular development and remodeling^5^, the vascular defects including reduced vessel diameters observed in mutant embryos and yolk sacs could be secondary. However, future studies using a real endothelial cell-specific Cre recombinase should be conducted to determine whether the deletion of HSP60 could directly cause vascular developmental defects.

Molecular chaperones are ubiquitously expressed and play various functions in erythroid development and maturation. Chaperones optimize protein synthesis in immature erythroblasts, which is particularly important in Diamond-Blackfan anemia, a disease in which haploinsufficiency of large or small ribosomal protein subunits leads to selective red cell aplasia^14^. In zebrafish, a loss-of-function mutation in the substrate binding domain of *Hsp9b* impairs the development of erythrocytes, granulocytes, and hematopoietic progenitors, probably resulting from mitochondrial dysfunction and increased oxidative stress^39^. Furthermore, molecular chaperones also promote erythropoiesis by regulating erythroblast survival against apoptotic stimuli. Maturing erythroblasts exhibit some degree of apoptosis including mitochondrial depolarization and transient activation of caspases^40,41^, which is also used physiologically to prevent excessive erythrocyte production. HSP70 has been shown to prevent active Caspase-3 from cleaving GATA1 and inducing apoptosis^15^. Increased apoptosis is also one of the essential features of ineffective erythropoiesis in Beta-thalassemias, a group of inherited blood disorders with reduced or absent synthesis of the beta chain of hemoglobin^42,43^. Here, we provided the first evidence, at least to our knowledge, that HSP60 also plays a critical role in regulating the survival of yolk sac erythrocytes in mouse embryonic development.

An increase of mitochondrial membrane permeability has been long recognized as one of the key events in both apoptotic and necrotic cell death. Opening of the mPTP leads to loss of mitochondrial membrane potential, mitochondrial swelling, and rupture of the outer mitochondrial membrane, resulting in the release of pro-caspases, cytochrome c, and apoptosis-inducing factor^44,45^. It has been proposed that inner membrane proteins, including the adenine nucleotide translocase, the mitochondrial phosphate carrier, the metalloprotease spastic paraplegia 7, and the F1F0-ATP synthase, form the opening pore, while outer membrane proteins, including VDAC, BAK, and BAX, play a modulatory role^44^. Ultimately, a general consensus has been reached that the matrix protein CypD plays an important role in regulating mPTP opening. Loss of CypD shifts the threshold for mPTP to a higher Ca^2+^ load, although mitochondria remain capable of undergoing permeability transition^28^. More importantly, genetic deletion of CypD in mice has proven protective against a series of different diseases including myocardial infarction, ischemia-reperfusion injury, muscular dystrophy, stroke, and Alzheimer’s disease^44^. A previous study in several tumor cell lines suggested that HSP60 may interact with CypD, and down-regulation of HSP60 reduces mitochondrial membrane potential and cell apoptosis^30^. HSP60 together with HSP10 is generally recognized to form the mitochondrial molecular chaperonin and promote protein folding. However, down-regulation of HSP60 in these tumor cells did not reduce mitochondrial CypD protein levels. It is more likely that HSP60 functions as the inhibitor CsA, preventing CypD from binding to mPTP and sensitizing the pore opening^30^. Consistently, we also found that deletion of HSP60 could reduce mitochondrial membrane potentials in yolk sac erythrocytes. More importantly, treating pregnant mice with CsA resulted in a decrease in cell apoptosis of yolk sac erythrocytes in mutant mice, further supporting the significant protective role of HSP60 in regulating mPTP opening and cell apoptosis in early erythropoiesis. However, CsA treatment only partially rescued cells from apoptosis and was not able to prevent fetal lethality. Therefore, a more comprehensive study should be performed in the future to determine whether deletion of HSP60 would affect other mitochondrial functions and mitochondrial protein homeostasis in erythrocytes.

Another interesting finding in our study is that VDAC expression in yolk sac erythrocytes was reduced due to the deletion of HSP60 and restored at least partially due to CsA treatment. In mammals, the VDAC protein family consists of three different isoforms including VDAC1, VDAC2, and VDAC3. Deletion of VDAC3 in mice did not result in embryonic lethality^33^. Deletion of VDAC1 in a mixed genetic background also did not cause embryonic lethality^46^. However, another study suggested that deletion of VDAC1 could lead to partial embryonic lethality^32^, suggesting that survivability of VDAC1 knockout embryos varies due to the genetic background. VDAC2 knockout mice have been reported to be embryonic lethal^34^, but detailed phenotypic analysis in mutant mice has not been well characterized. Although VDAC1 is generally considered the most abundant isoform of VDAC in various tissues^31^, our immunostaining results using the isoform-specific antibodies suggested that VDAC2, but not VDAC1, is preferably expressed in yolk sac erythrocytes. VDAC may regulate the transport of metabolites and ions including Ca^2+^, energy production, and lipid metabolism^47^. Functionally, overexpression of VDAC1 has been shown to induce apoptotic cell death in various cell types, whereas reducing VDAC1 expression prevented cell apoptosis and Bax activation^22^. It has also been shown that cells deficient in VDAC2, but not VDAC1, exhibit enhanced BAK oligomerization, and are more susceptible to apoptotic death. In addition, overexpression of VDAC2 reduces the mitochondrial apoptotic pathway^34,48^. Therefore, down-regulation of VDAC2 expression in mutant erythrocytes might contribute to the increase of cell apoptosis after HSP60 deletion. However, the molecular mechanisms underlying how VDAC2 expression is regulated by HSP60 should be determined in the future. Our study demonstrates that HSP60 plays an important role in yolk sac erythropoiesis. Deletion of HSP60 increased cell apoptosis partially via a mPTP-dependent mechanism, and eventually resulted in reduced numbers of erythrocytes, anemia, and embryonic lethality.

## Materials and methods

### Mice

The *Hsp60*^f/f^ mice were generated as previously described^49^. The *Tie2-Cre*^+^ mice, which express the Cre recombinase under the control of the mouse endothelial-specific receptor tyrosine kinase (Tie2) promoter^25,50^, are commercially obtained (The Jackson Laboratory). The *Hsp60*^f/f^ mice were first crossed with the *Tie2-Cre*^+^ mice to generate *Tie2-Cre*^+^*Hsp60*^f/+^ mice. The male *Tie2-Cre*^+^*Hsp60*^f/+^ mice were subsequently used to cross with female *Hsp60*^f/f^ mice to generate *Tie2-Cre*^+^*Hsp60*^f/f^ (HSP60^CKO^) mice. The littermates, *Tie2-Cre*^−^*Hsp60*^f/+^ and *Tie2-Cre*^−^*Hsp60*^f/f^, were used as control mice.

### DNA analysis

Genomic DNA was extracted from mouse tails as previously described^51^, and polymerase chain reaction was used to genotype the offspring using the following gene-specific primers (from 5’ to 3’): *Tie2-Cre* (forward, CCCTGTGCTCAGACAGAAATGAGA; reverse, CGCATAACCAGTGAAACAGCATTGC), *Hsp60* (forward, TGGGTCAAGTACTTTTATCCCCTA; reverse, GGGAAGGCTAAGACCTACTCATT).

### Morphological analysis

Embryos were collected after a timed mating using a 12-h light/dark cycle, with noon on the day of discovering a vaginal plug defined as E0.5. Embryos and yolk sacs were dissected from the maternal decidua under a Leica MZ6 dissecting light microscope and photographed, as previously described^52^.

### Whole-mount PECAM staining

Embryos were collected in ice cold phosphate buffered saline (PBS), and fixed in 4% paraformaldehyde (PFA) diluted in PBS overnight at 4 °C. After fixation, embryos were dehydrated sequentially through a methanol series (25, 50, 75, and 100% methanol) for 15 min at room temperature. The embryos were next quenched with 5% hydrogen peroxide, and then washed twice with 100% methanol at room temperature. Afterwards, the embryos were rehydrated through 75, 50, and 25% methanol and PBS twice. Subsequently, the embryos were incubated with the blocking solution for several hours at room temperature and then incubated with the primary CD31 or PECAM antibody (BD Pharmingen, catalog no. 550274) at 4 °C overnight. Samples were washed and incubated with HRP-conjugated goat anti-rat IgG (Zsbio, catalog no. ZB2307) at 4 °C overnight. Finally, 3’,3’-diaminobenzidine was used to develop the color, and pictures were acquired using a stereomicroscope.

### Whole mount Immunofluorescence staining

Extraembryonic yolk sacs were dissected and fixed with 4% PFA diluted in PBS at 4 °C overnight, and whole mount immunostaining was performed using a modified protocol as previously described^53,54^. Yolk sacs were briefly washed with PBS after fixation, and incubated with the blocking buffer (2% bovine serum albumin, 5% horse serum in PBS containing 0.2% Triton X-100). The samples were then incubated with primary antibodies at 4 °C overnight. Subsequently, the samples were washed and incubated with Alexa-conjugated secondary antibodies for 1 h at room temperature. Finally, the samples were flat-mounted using Dako Fluorescent Mounting Media, and pictures were acquired under an Olympus 1 × 73 Fluorescence Microscopy or Nikon A1R confocal microscope. The numbers of GATA1 positive cells at E8.5 and E9.0 were counted from the whole yolk sacs, and the numbers of cleaved-Caspase 3 positive, phosphorylated Histone 3 positive cells were counted in at least three randomly chosen visual fields. The primary antibodies against CD31 (BD Pharmingen, catalog no. 550274), GATA1 (Santa Cruz Biotechnology, catalog no. sc-265), HSP60 (Santa Cruz Biotechnology, catalog no. 13966), LYVE1 (Abcam, catalog no. ab14917), phospho-histone 3 (Sigma, catalog no. 06-570), Cleaved Caspase-3 (Cell Signaling Technology, catalog no. 9661), Cyt C (Cell Signaling Technology, catalog no. 4280), CypD (Thermofisher, catalog no. PA1-028), NIMP (Santa Cruz Biotechnology, catalog no. sc-514049), COX4 (Santa Cruz Biotechnology, catalog no. sc-19998), VDAC1/2/3 (Santa Cruz Biotechnology, catalog no. sc-98708), VDAC1 (Santa Cruz Biotechnology, catalog no. sc-390996), and VDAC2 (Proteintech, catalog no.11663-1-AP), were commercially purchased.

### Measurement of mitochondrial membrane potential using flow cytometry

Yolk sacs were first isolated and incubated with 1 × PBS containing 0.5% collagenase II (Worthington, catalog no. LS004177) and 10% FBS for 10 min at 37 °C. The single-cell suspensions were then prepared by passing the tissues through 100 μm nylon. Subsequently, the cells were incubated with tetramethylrhodamine (TMRM; 100 nM; Invitrogen, catalog no. 13461) for 30 min at 37 °C, washed, and then incubated with PerCP-conjugated Ter119 (Biolegend, catalog no. 116225). The dead cells were excluded by 7-AAD (Biolegend, catalog no. 420403). Flow cytometry was performed on BD FACSCanto II (BD Biosciences) and data was analyzed with FlowJo (Tree Star) as previously described^55^.

### Benzidine staining

The embryos with yolk sacs were incubated with 12% glacial acetic acid containing 0.4% benzidine (Sigma, B-3503) for 5 min at room temperature. After that, hydrogen peroxide was added to a final concentration of 0.3%, and the tissues were incubated for another 10–15 min until the color developed. The tissues were then washed with 12% acetic acid, fixed in methanol, and imaged using a stereomicroscope.

### Chemical injection

Ac-DEVD-CHO (Selleck, catalog no. S7901) and CsA (Sangon, catalog no. A600352) were dissolved in DMSO and 50% ethanol, respectively. Pregnant mice with the embryos at E7.5 were injected intraperitoneally with 20 mg/kg CsA or 10 mg/kg Ac-DEVD-CHO, and the embryos were then dissected and examined at E9.0.

### Statistics

Scatter diagrams were drawn using Graphpad Prism 5 as previously described^56^. Statistical analysis was performed using a 2-tailed, unpaired Student’s *t* test or a two-way ANOVA with a Bonferroni post-hoc test for multiple comparisons when the assumptions of normality assessed with the Shapiro-Wilks test are met using SPSS Statistics. The non-parametric Mann–Whiney *U* test was used for independent (unpaired) groups which are non-normally distributed. For each experiment, at least three independent samples per group were examined. All data represents the mean ± SEM (error bars). *P* *<* 0.05 was considered statistically significant.

### Study approval

All animal care and use procedures were approved by the Animal Care and Use Committee at Peking University Shenzhen Graduate School (Shenzhen, China). Periodic review of procedures was performed, and amendments were made as needed.

## Supplementary information

Supplemental Figure legends

supplemental figure 1

supplemental figure 2

supplemental figure 3

supplemental figure 4

supplemental figure 5

supplemental figure 6
