# Supplementary material for: Heat shock protein 60 regulates yolk sac erythropoiesis in mice
Source: Cell Death Dis. 2019 Oct 10;10(10):766. doi: 10.1038/s41419-019-2014-2 (PMC6786998; doi:10.1038/s41419-019-2014-2)
Supplement: Supplementary file 1 — Supplemental Figure legends [file 41419_2019_2014_MOESM1_ESM.docx]

**Supplemental Figure legends**

**Supplemental Figure 1. Expression of VDAC, Cytochrome C, and HSP60 in yolk sac erythrocytes.** Yolk sacs were isolated from wildtype embryos at E8.5, and whole-mount immunofluorescence staining was performed to examine the expression of VDAC (**a**), Cytochrome C (Cyt C, **b**) and HSP60 (**c**), respectively. GATA1 was used to indicate the erythrocytes in yolk sacs. Data are representative of at least three independent experiments. Scale bar, 20 μm.

**Supplemental Figure 2. Generation and characterization of tissue-specific HSP60 knockout mice by Tie2-Cre.** (**a**) Schematic diagram of the mouse breeding strategy used to generate *Tie2-Cre*^+^*Hsp60*^f/f^ (HSP60^CKO^) mice. The littermates *Tie2-Cre*^-^*Hsp60*^f/+^ and *Tie2-Cre*^-^*Hsp60*^f/f^ mice were used as the control mice. (**b**) Polymerase chain reaction (PCR) was used to identify wildtype, *Hsp60* floxed, and *Tie2-Cre* alleles. The PCR products in *Hsp60* floxed alleles are around 50 bp longer than those in wildtype alleles. P, positive control. N, wildtype control.

**Supplemental Figure 3. Expression of VDAC1 and VDAC2 in yolk sac erythrocytes.** (**a**) Whole-mount immunofluorescence staining of GATA1 and VDAC1 in control and HSP60^CKO^ yolk sacs at E9.0, indicating that VDAC1 was highly expressed in GATA1 negative cells. Data are representative of at least three independent experiments. Scale bar, 20 μm. (**b**) Whole-mount immunofluorescence staining of GATA1 and VDAC2 in control and HSP60^CKO^ yolk sacs at E9.0. VDAC2 had a clear perinuclear expression pattern in GATA1 positive erythrocytes, and deletion of HSP60 could abolish VDAC2 expression in yolk sac erythrocytes. Data are representative of at least three independent experiments. Scale bar, 20 μm.

**Supplemental Figure 4. Expression of CypD, NIMP, and COX4 in control and HSP60^CKO^ yolk sacs**. Whole-mount co-immunostaining of GATA1 with CypD (a), NIMP (b), and COX4 (c) in control and HSP60^CKO^ yolk sacs at E9.0, respectively. Data are representative of at least three independent experiments. Scale bar, 20 μm.

**Supplemental Figure 5. Caspase inhibitor reduced cell apoptosis induced by HSP60 deletion in mutant erythrocytes.** (a) Schematic diagram showing the procedure of the rescue experiment. Pregnant mice were intraperitoneally injected with Ac-DEVD-CHO at E7.5. The embryos were dissected at E9.0, and immunofluorescence staining was then performed. (b) Immunofluorescence staining of GATA1 and cleaved-Caspase 3 (cl-C3) in control and mutant yolk sacs treated with (+ Ac-DEVD-CHO) and without (- Ac-DEVD-CHO) Ac-DEVD-CHO at E9.0. Scale bar, 100 μm. (c) Statistical analysis showing that Ac-DEVD-CHO treatment could significantly reduce cell apoptosis of erythrocytes in mutant yolk sacs. n = 4 mice per group. Data represent mean ± SEM. Significance was determined using the 2-way ANOVA analysis with a Bonferroni post-hoc test. **p* < 0.05, ***p* < 0.001, ****p* < 0.001.

**Supplemental Figure 6. CsA treatment partially restored VDAC2 expression in HSP60^CKO^ yolk sac erythrocytes.** Immunofluorescence staining of GATA1 and VDAC2 in yolk sacs of E9.0 control and HSP60^CKO^ embryos isolated from pregnant mice untreated (-CsA) or treated with CsA (+CsA). A clear perinuclear staining of VDAC2 was detectable in HSP60^CKO^ yolk sac erythrocytes at E9.0 when the pregnant mice were treated with CsA. Data are representative of at least three independent experiments. Scale bar, 20 μm.
