# Supplementary figures and images for: Heat shock protein 60 regulates yolk sac erythropoiesis in mice
Source: Cell Death Dis. 2019 Oct 10;10(10):766. doi: 10.1038/s41419-019-2014-2 (PMC6786998; doi:10.1038/s41419-019-2014-2)

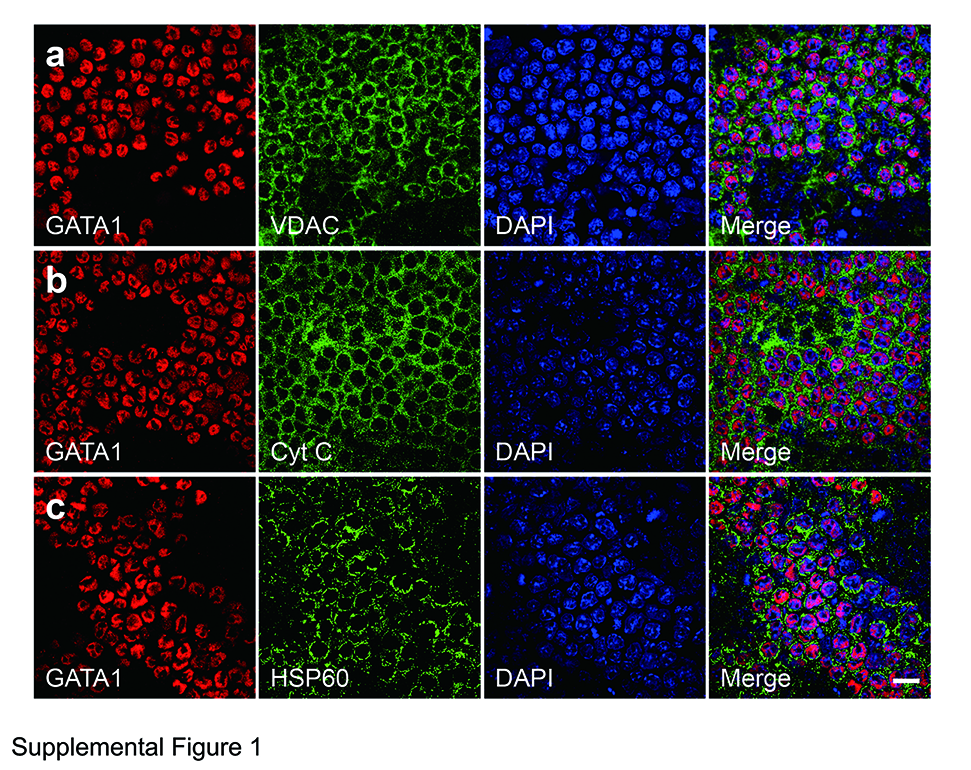

Supplement: Supplementary file 2 — supplemental figure 1 [file 41419_2019_2014_MOESM2_ESM.tif]

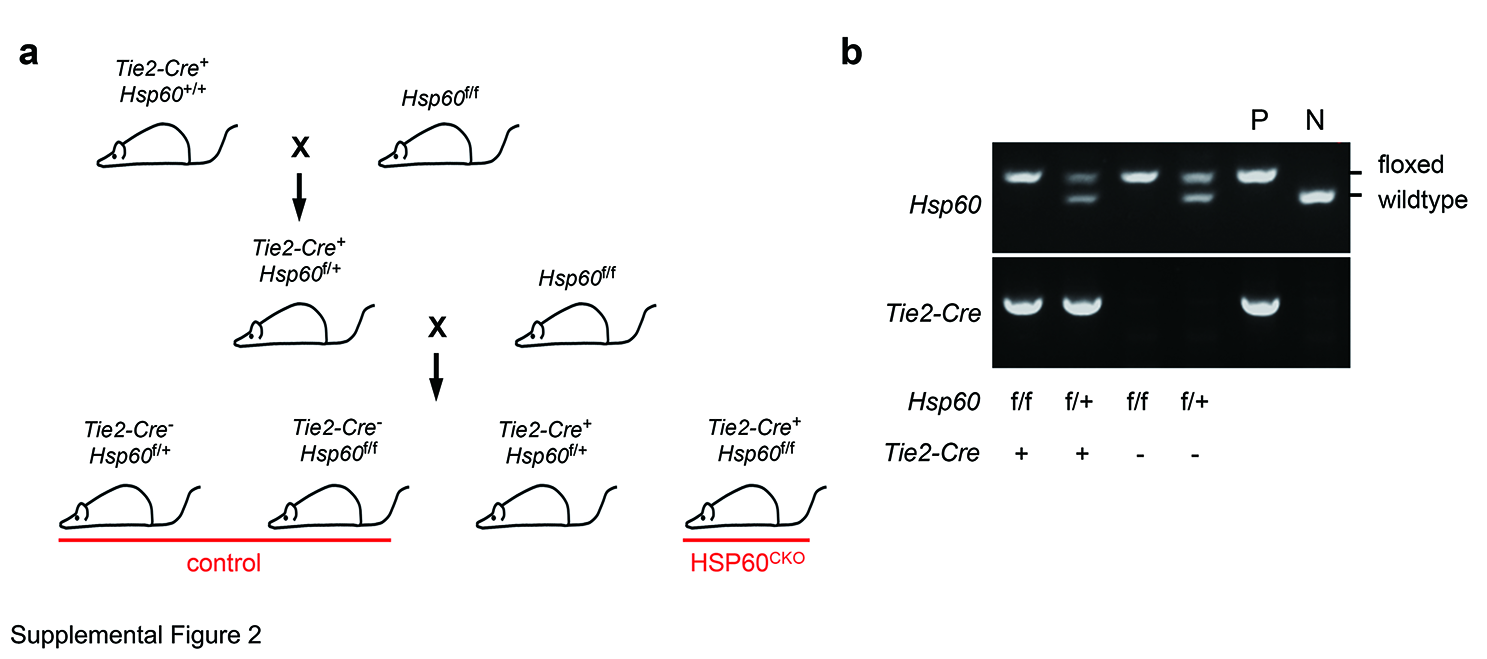

Supplement: Supplementary file 3 — supplemental figure 2 [file 41419_2019_2014_MOESM3_ESM.tif]

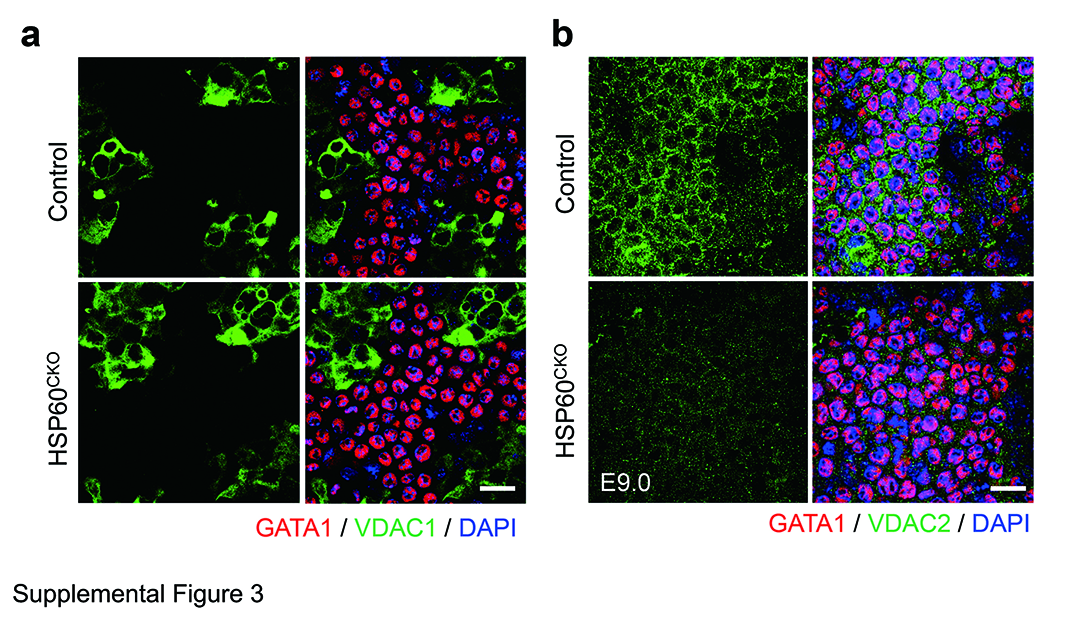

Supplement: Supplementary file 4 — supplemental figure 3 [file 41419_2019_2014_MOESM4_ESM.tif]

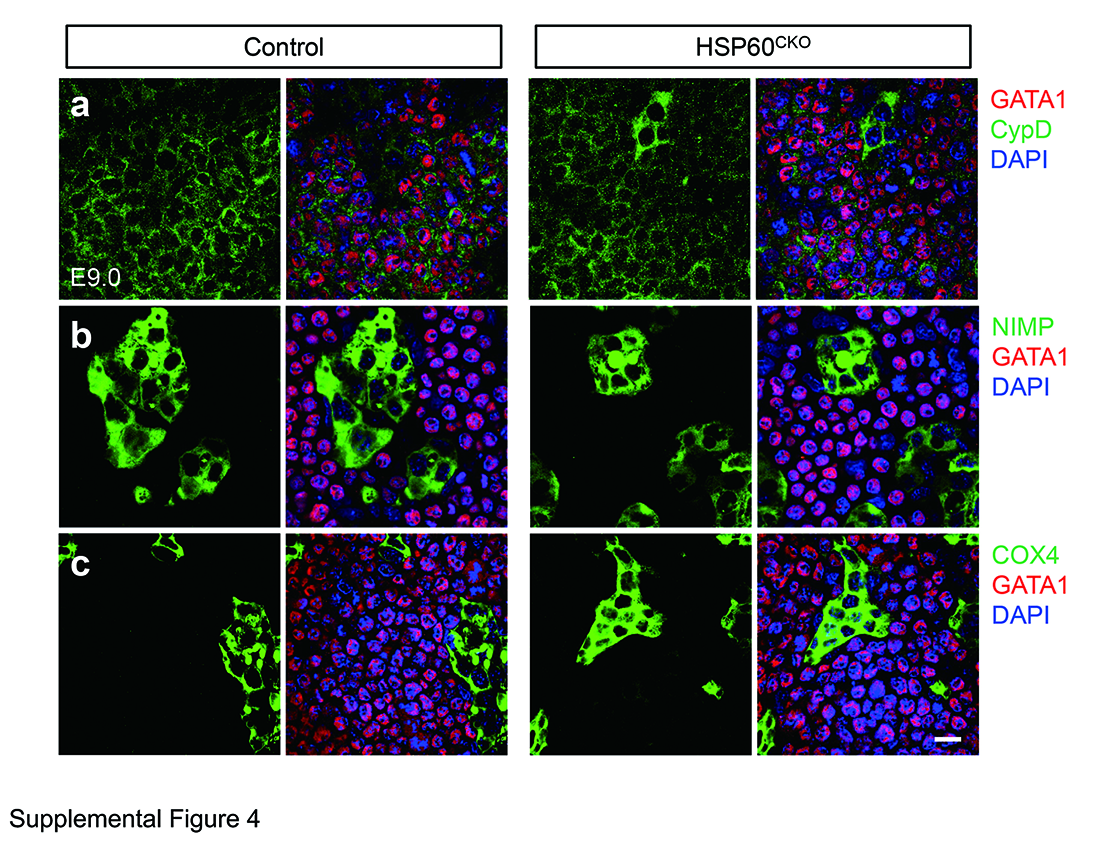

Supplement: Supplementary file 5 — supplemental figure 4 [file 41419_2019_2014_MOESM5_ESM.tif]

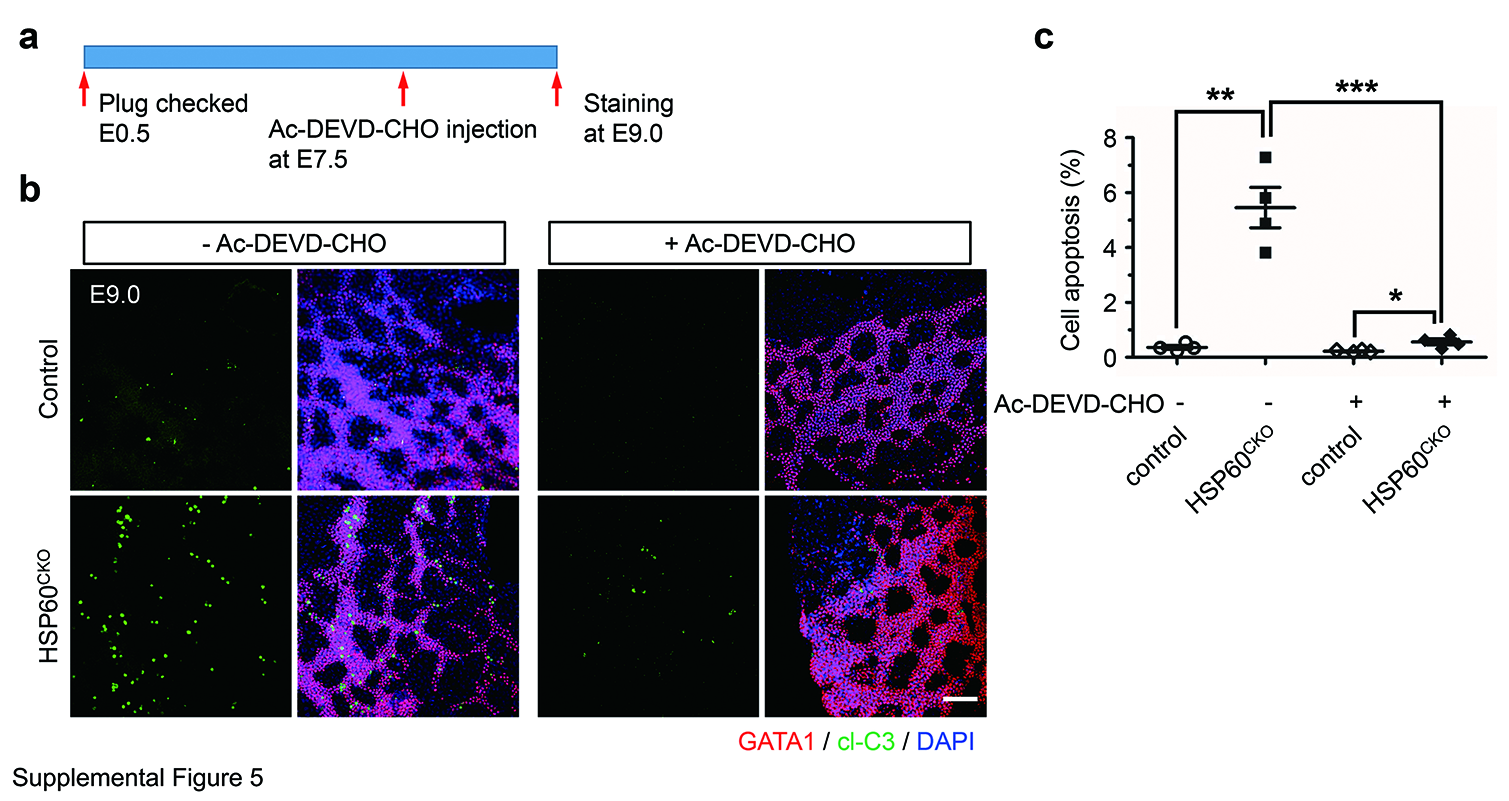

Supplement: Supplementary file 6 — supplemental figure 5 [file 41419_2019_2014_MOESM6_ESM.tif]

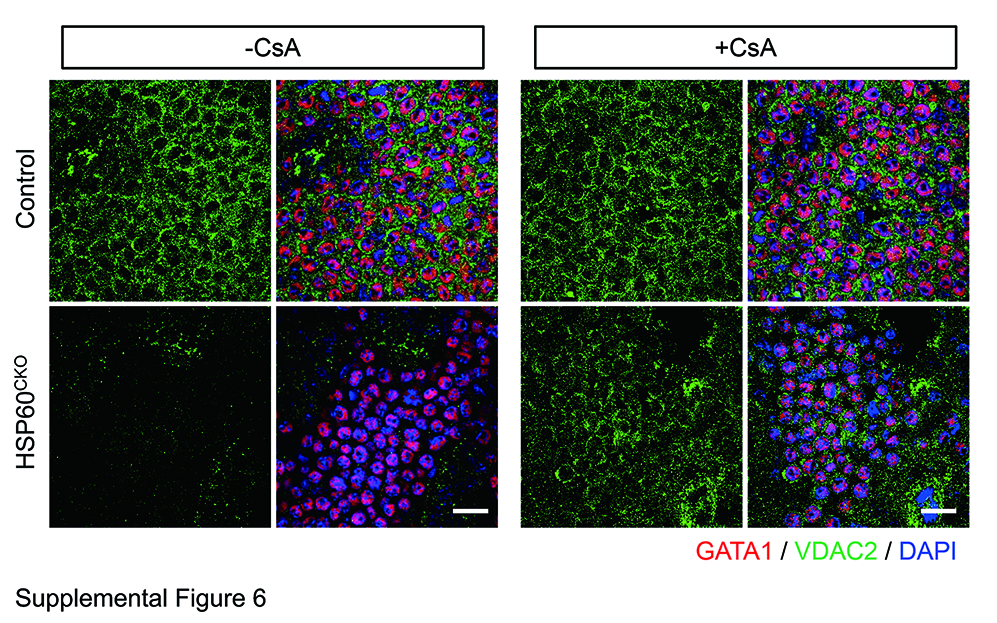

Supplement: Supplementary file 7 — supplemental figure 6 [file 41419_2019_2014_MOESM7_ESM.tif]
